# Lymphocutaneous Sporotrichosis during Treatment with Anti-TNF-Alpha Monotherapy

**DOI:** 10.1155/2015/614504

**Published:** 2015-02-10

**Authors:** Francesco Ursini, Emilio Russo, Christian Leporini, Marilena Calabria, Caterina Bruno, Cesare Tripolino, Saverio Naty, Rosa Daniela Grembiale

**Affiliations:** University “Magna Græcia” of Catanzaro, 88100 Catanzaro, Italy

## Abstract

Sporotrichosis is an infectious disease caused by *Sporothrix schenckii*, a dimorphic fungus isolated for the first time in 1896 by Benjamin Schenck from a 36-year-old male patient presenting lesions on the right hand and arm. The infection generally occurs by traumatic inoculation of soil, plants, and organic matter contaminated with the fungus. Different clinical syndromes are described as a direct consequence of *S. schenckii* infection, including lymphocutaneous and disseminated forms, although extracutaneous presentations are reported most frequently in AIDS patients. Here we describe the case of a 57-year-old Caucasian male diagnosed in 2004 with ankylosing spondylitis under stable treatment with adalimumab monotherapy (40 mg every other week). During a routine follow-up visit in March 2013, he presented with multiple nodular lesions arranged in a linear fashion along the left hand and forearm. After diagnostic aspiration of the lesions, lymphocutaneous sporotrichosis was diagnosed and appropriate therapy started.

## 1. Introduction

Sporotrichosis is an infectious disease caused by* Sporothrix schenckii*, a dimorphic fungus isolated for the first time in 1896 by Benjamin Schenck from a 36-year-old male patient presenting lesions on the right hand and arm. The infection generally occurs by traumatic inoculation of soil, plants, and organic matter contaminated with the fungus. Different clinical syndromes are described as a direct consequence of* S. schenckii* infection, including lymphocutaneous and disseminated forms, although extracutaneous presentations are reported most frequently in AIDS patients [1].

## 2. Case Presentation

Our patient was a 57-year-old Caucasian male diagnosed in 2004 with ankylosing spondylitis. Since 2007, he was under stable treatment with adalimumab monotherapy (40 mg every other week).

**Figure 1:**
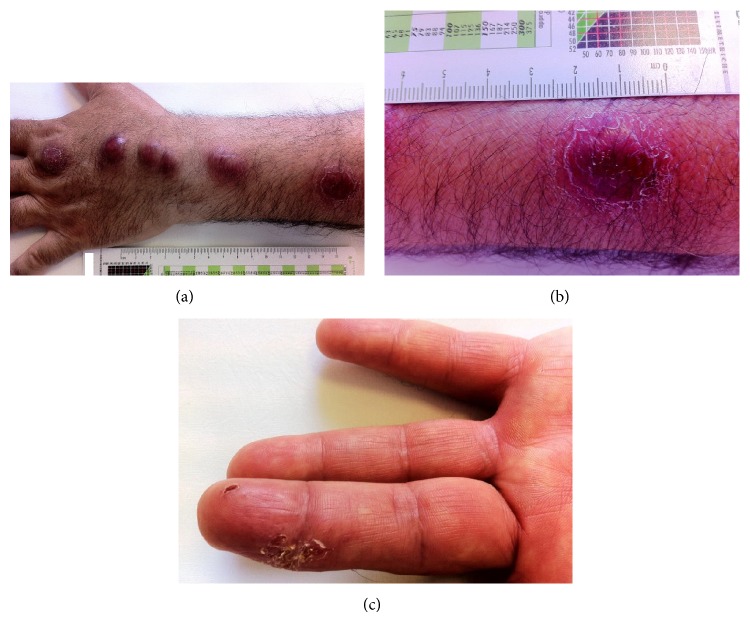
Multiple nodular lesions arranged in a linear fashion along the left hand and forearm of the patient (a). Detail of the nodules (b). Probable site of fungus inoculation, represented by a small ulcer on the fingertip of the third digit (c).

The patient came to our observation during a routine follow-up visit in March 2013. In that occasion he presented with multiple nodular lesions arranged in a linear fashion along the left hand and forearm (Figure 1(a)). The nodules appeared reddish in color, not painful, and not ulcerated, with the maximum diameter of 2.2 cm (Figure 1(b)). The patient was afebrile and his general condition was good.

The clinical presentation suggested* S. schenckii* infection, and we have been able to identify the probable site of fungus inoculation, represented by a small ulcer on the fingertip of the third digit (Figure 1(c)). The patient was not able to remember exactly how and when this injury had arisen. However, the most likely hypothesis is that of an unnoticed puncture with rose thorn, because these flowers are abundant in the home garden of the patient.

Laboratory testing was immediately performed, showing only a mild elevation of alanine aminotransferase (59 IU/L), aspartate aminotransferase (40 IU/L), and creatine phosphokinase (313 IU/L). Complete blood count, erythrocyte sedimentation rate, C-reactive protein, and procalcitonin were in the normal range.

In the following days, an ultrasound evaluation of subcutaneous nodules was performed. The lesions appeared as well-defined hypoanechoic subcutaneous nodules with internal debris. Finally, diagnostic needle aspiration of the biggest nodule was performed.* Sporothrix schenckii* was isolated from culture of the collected material.

The final diagnosis was of cutaneous sporotrichosis with nodular lymphangitic spread. Adalimumab was discontinued and appropriate treatment with oral itraconazole 200 mg/daily was subsequently started. After 3 months of treatment the patient improved significantly but after 9 months sequelae of the lesions were still present.

## 3. Discussion

Sporotrichosis has a higher prevalence in tropical and temperate zones and appears only sporadically in Europe [1]. In Italy, only 58 cases of cutaneous sporotrichosis have been described until 1992 [2] and a few others have been added to the literature in the last 20 years. To the best of our knowledge, only two cases of sporotrichosis in inflammatory arthritis patients treated with biologics have been described to date. The first was reported in 2003 by Gottlieb et al. [3] in a patient treated with multiple disease-modifying antirheumatic drugs (DMARDs) including infliximab and etanercept; the latter was reported by Yamaguchi et al. [4] in 2012 in a patient sequentially treated with different medications including tocilizumab. Thus, differently from our patient, both aforementioned cases received multiple immunosuppressants; conversely our patient was under stable treatment with adalimumab monotherapy since 6 years.

Adalimumab was approved by the European Medicines Agency (EMA) in September 2003 for the treatment of moderate to severe rheumatoid arthritis and subsequently for the treatment of ankylosing spondylitis, psoriasis and/or psoriatic arthritis, juvenile idiopathic arthritis, and Crohn's disease. This agent is a fully recombinant human immunoglobulin G1 monoclonal antibody that specifically binds with high affinity to human tumor necrosis factor- (TNF-) alpha, thereby inhibiting its interaction with TNF receptors. TNF-alpha exerts modulatory effects on several aspects of cellular and humoral immunity. In particular, it plays a central role in the host defense against bacteria, viruses, and parasites [5]. Accordingly, serious fungal, mycobacterial, and bacterial infections have been reported to be associated with treatment with TNF-alpha antagonists [6]; however, the different pharmacodynamic and pharmacokinetic properties of these agents might change their associated risks of infections [7].

Fungal infections associated with TNF-alpha blockade have been reported occasionally, and they are limited to isolated cases or small series of patients [8]. However, published information about several fungal infections was reported without further details about the clinical course of each individual patient [9]. Therefore, some of the cases reported by different authors could overlap. In addition, published clinical trials of anti-TNF-alpha agents are biased from several design limitations (e.g., small number of patients and short follow-up) [10, 11]. In light of these considerations, the exact incidence of fungal infections complicating TNF-alpha antagonist therapy remains challenging to estimate [8].

In a recent review, Tsiodras et al. [8] reported 281 cases of fungal infections associated with TNF-alpha inhibition. Of these cases, 226 (80%) were associated with infliximab, 44 (16%) with etanercept, and 11 (4%) with adalimumab. Fungal infections associated with infliximab occurred a median of 55 days after initiation of therapy and 3 infusions of the medication, whereas those associated with etanercept occurred a median of 144 days after initiation of therapy. The median age of patients was 58 years, and 62% were male. The use of at least 1 other immunosuppressant drug (e.g., corticosteroids and methotrexate) was reported during the course of the fungal infection in 102 (98%) of the 104 patients for whom data were available. The most frequently observed infections were histoplasmosis (*n* = 84 (30%)), candidiasis (*n* = 64 (23%)), and aspergillosis (*n* = 64 (23%)) [8]. Only one case of sporotrichosis associated with TNF-alpha blockade was described in a patient receiving etanercept and infliximab as well as corticosteroids and methotrexate. Following this treatment, he developed cutaneous and osteoarticular sporotrichosis which was treated with amphotericin B and itraconazole for a prolonged period [3].

The immunological mechanisms involved in prevention and control of* S. schenckii* infection are still not completely understood.

Abnormal TNF-alpha regulation seems to characterize* S. schenckii* infection in mice [12], suggesting a mechanism by which TNF-alpha antagonists might predispose a person to infection by* S. schenckii* and consequent sporotrichosis. According to experimental data obtained from mouse models, acquired immunity against* S. schenckii* is mediated mainly by macrophages activated by CD4+ T cells expressing IFN-gamma, TNF-alpha, and interleukin-10 [13]. Moreover, recent evidence suggests a pivotal role of macrophage Toll-like receptor 2 (TLR-2), a TNF-alpha-regulated receptor, in the recognition of* S. schenckii* [14]. Thus, therapeutic blockade of TNF-alpha signaling could result in reduced expression of TLR-2 on macrophages, leading to impaired recognition of the fungus. In addition, opsonization has been demonstrated to enhance TNF-alpha production and fungus killing by macrophages in experimental sporotrichosis [15].

In conclusion, our report describes* S. schenckii*-related infectious complication of adalimumab, which was not related to the concurrent use traditional immunosuppressive medications, as reported in other cases of sporotrichosis in inflammatory arthritis patients. Therefore, this case suggests that even in patients with anti-TNF-alpha monotherapy the index of suspicion for opportunistic fungal infections should be maintained high.
